# Syngeneic Mouse Models for Pre-Clinical Evaluation of CAR T Cells

**DOI:** 10.3390/cancers16183186

**Published:** 2024-09-18

**Authors:** Eman N. Ahmed, Lauren C. Cutmore, John F. Marshall

**Affiliations:** 1 Centre for Tumour Biology, Barts Cancer Institute, Queen Mary University of London, London EC1M 6BQ, UKlauren.cutmore@nih.gov (L.C.C.); 2 College of Medicine, Alfaisal University, Riyadh 11533, Saudi Arabia

**Keywords:** CAR T cells, adoptive cellular therapy, solid tumors, immunotherapy, syngeneic models, combination therapy

## Abstract

**Simple Summary:**

CAR T cells are a type of immune cell that is genetically engineered to better recognize and attack cancer cells. Before this treatment can be used to treat humans, it undergoes testing in pre-clinical models. Most of these models use mice that do not have an immune system, which means that these models may not accurately recapitulate CAR T cell actions in humans. This review focuses on how using syngeneic mouse models that have a functional immune system can result in better pre-clinical assessment of CAR T cells.

**Abstract:**

Chimeric antigen receptor (CAR) T cells have revolutionized the treatment of hematological malignancies. Unfortunately, this improvement has yet to be translated into the solid tumor field. Current immunodeficient models used in pre-clinical testing often overestimate the efficacy of CAR T cell therapy as they fail to recapitulate the immunosuppressive tumor microenvironment characteristic of solid tumors. As CAR T cell monotherapy is unlikely to be curative for many solid tumors, combination therapies must be investigated, for example, stromal remodeling agents and immunomodulators. The evaluation of these combination therapies requires a fully immunocompetent mouse model in order to recapitulate the interaction between the host’s immune system and the CAR T cells. This review will discuss the need for improved immunocompetent murine models for the pre-clinical evaluation of CAR T cells, the current use of such models and future directions.

## 1. Introduction

Adoptive cellular therapy has resulted in advancements in treating multiple malignancies, particularly hematological malignancies and more recently solid tumors. Chimeric antigen receptor (CAR)-engineered T cell therapy is based on the principle of manipulating T cells in vitro such that cells express a chimeric antigen receptor that selectively recognizes tumor cells, which are then re-introduced into the patient. CARs are engineered by combining an antigen-binding domain to a T cell signaling molecule (CD3ζ), which allows the CAR to mimic endogenous TCR-mediated activation without any of the effects of classic MHC restriction. These CAR T cells can be designed to target practically any surface antigen expressed on cancer cells, unlike physiological TCRs, which are restricted by thymic selection. Additionally, the CAR platform has the ability to include costimulatory molecules (such as CD28 or 4-1BB) in the signaling domain to improve T cell proliferation, cytokine production and tumor cell lysis (reviewed in [1,2,3]). Currently, four CD19-targeting and two BCMA-targeting CAR T cells are licensed by the United States Food and Drug Administration (FDA) for the treatment of hematological malignancies [4].

Prior to use in patients, CAR T cells are evaluated in mouse models to look for tumor eradication and improvements in survival and toxicities. However, the effectiveness of CAR T cells needs to be revised in terms of appropriate mouse model selection depending on the aims of the study. This review will shed light on why evaluating CAR T cells using syngeneic mouse models will more accurately recapitulate patient responses compared to immunocompromised models. We will also discuss recent models (Supplementary Table S1) and reflect on the requirements for models that will help us generate more effective CAR T cell therapies for solid and hematological malignancies in patients.

## 2. Current Models

Multiple pre-clinical models have been used to assess CAR T cells; a comprehensive review of these models has been performed by Duncan et al. [5]. Murine models used to test CAR T cells in vivo can be broadly categorized into those using human CAR T cells and those using murine CAR T cells. Pre-clinical models that test human CAR T cells are obligated to use immunodeficient mouse models to ensure engraftment of the human tumor cells and to prevent rejection of the human T cells. The most common models used in CAR T cell research are NODShi.Cg-Prkdc^scid^Il2rg^tm1Sug^ (NOG) and NOD.Cg-Prkdc^scid^ Il2rg^tm1Wjl^ (NSG) mice, which are severely immunodeficient. These models lack an adaptive immune system, are deficient in T cells, NK cells and B cells, and also have reduced innate immunity [6,7].

The most basic of these immunodeficient models are xenograft models, where immortalized human cell lines are introduced into the mouse to form tumors (Table 1). These are then treated using human CAR T cells. A more clinically relevant variation of these models is the patient-derived xenograft. In this model, fragments from patient tumors are engrafted into the animals; this includes the human stroma associated with the tumor [8]. Another type of murine model used in pre-clinical CAR T testing consists of humanized mice, which are immunodeficient mice whose immune cells have been replaced with human immune cells (Table 1) [9]. While this model overcomes some of the limitations seen with immunodeficient models and can provide a more complete view of the interplay between the immune system and the tumor, its extremely high cost (financially and in time) of production makes its routine use for testing human CAR T cells prohibitive. Although such studies have merit, they fail to recapitulate the effects on therapy imposed by the strong immunosuppression caused by the innate and adaptive immune cells in the tumor microenvironment, which inhibits CAR T cell efficacy.

Using murine CAR T cells in immunocompetent mice allows for a better understanding of the interplay between the host immune system and the CAR T cells. This is particularly important when developing CAR T cells for the treatment of solid tumors with highly immunosuppressive microenvironments [10]. The most basic of these syngeneic models uses murine cell lines engrafted into the mice to form tumors (Table 1) [11]. In addition to administering tumor cells directly to the mice, it is also possible to use spontaneous tumor models. These models are genetically engineered to have mutations that result in tumor formation. These models may better recapitulate the stroma as they allow for long-term tumor development. However, these are expensive to develop and can be time-consuming to use. There are also often inconsistencies in the development of the tumors, which leads to further difficulty in their use. Chemical carcinogens or radiation can also be used to induce mutations in the mice. This results in high mutational rates and initiation costs are low. However, the resulting tumors will have random mutations and consistency between animals may be hampered [12]. Transgenic mice are another syngeneic model that can be utilized. This model utilizes genetic knock-in of human antigens into the murine cells [11]. The tumors are then treated with murine T cells that express a CAR targeting the human antigen (Table S1).

Thus, using mouse CAR T cells within syngeneic mice can be beneficial in the development of strategies to solve immunosuppression issues and to develop more efficacious CAR T cells. A complete list of syngeneic mouse models that have been used in the pre-clinical testing of CAR T cells is available in Supplementary Table S1.

## 3. Changes Needed in Murine CAR Structure

A chimeric antigen receptor (CAR) construct is a genetically engineered receptor made by combining multiple domains to produce a single functional receptor (reviewed previously [3]). Briefly, the CAR is expressed on the surface of a T cell and binds to the target antigen on cancer cells to initiate a cytotoxic reaction without the help of MHC molecules. The molecular composition of CARs contains three domains: the extracellular, transmembrane and intracellular domain. The extracellular domain is the part of the CAR that binds to the target antigen. This is often a single-chain variable fragment (scFv) derived from an antibody against a tumor-associated antigen. This is followed by the transmembrane domain, and then the intracellular domain made from activating regions of a TCR. The composition of the intracellular domain depends on the generation of CAR being studied. First-generation CARs only contain a signaling domain, usually CD3ζ, which is responsible for initiating the cytotoxic signals within the T cell [13]. Second- and third-generation CARs include one or two costimulatory domains, respectively, which help with the persistence and activity of the CAR T cells. Commonly used costimulatory domains include CD28 and 4-1BB [14,15].

In order for CAR T cells to be used in an immunocompetent mouse model, it is necessary for the CAR construct to be redesigned to have fully murine components. If the conventional human components are used, they may result in the CAR T cell being rejected and destroyed by the host immune system. The human CD3ζ domain used in many human CAR constructs has a murine homolog with 84.07% sequence homology [16,17]. For costimulatory domains, CD28 has 68.90% homology and 4-1BB has 57.03% homology between the two species [18,19]. These differences illustrate the need for the redesign of the CAR. Additional modifications to the CAR structure have also been established, which will be discussed in more detail throughout this review.

The most effective method for CAR integration into the genome also varies between human and murine T cells. Lentiviral vectors are an effective and popular method of gene integration in human T cells; however, they do not seem to have the same efficacy in murine cells [20,21]. Retroviral vectors have proven to be more effective for the stable integration of genes into murine T cells [22].

## 4. Preconditioning

There is increasing evidence (discussed below) that successful adoptive therapies are dependent on preconditioning with immunosuppressive drugs, such as cyclophosphamide or irradiation, to enhance treatment efficacy [23]. There are multiple reasons why preconditioning to depleted immune cells is beneficial for CAR T cell activity. Firstly, it can reduce immunosuppressive cells, such as regulatory T cells and myeloid-derived suppressor cells, that can inhibit the cytotoxic potential of CAR T cells. Secondly, it can remove the endogenous T cells, which act as a “cytokine sink” [24]. The removal of these cells results in higher levels of IL-2, IL-7 and IL-15 in the body, which can be utilized by the CAR T cells and improve their effector function [25,26].

Additionally, preconditioning can directly modulate the tumor cells to increase the expression of costimulatory molecules and reduce the expression of indoleamine 2,3-dioxygenase (IDO). These changes make the tumor cells more sensitive to CAR T cell-mediated killing [27]. Preconditioning with radiotherapy has been shown to enhance tumor clearance in solid tumor models. It was demonstrated that the irradiation of tumor cells could upregulate the expression of the target antigen, as well as increase the expression of molecules that can promote T cell infiltration, such as intracellular adhesion molecule-1 (ICAM-1) or FAS, leading to greater CAR T cell infiltration within the tumor [28].

Although lymphodepletive preconditioning does not negatively affect the majority of patients, the toxicity of chemotherapeutic agents poses a threat for those of low performances status, rendering them unable to receive CAR T cell therapy [29]. The effects of preconditioning are not evaluated in immunodeficient models as there is no need to further lymphodeplete these mice; hence, these studies are not reflective of clinical settings as patients often receive lymphodepletive preconditioning during treatment [23].

Lymphodepletive preconditioning administered prior to CAR T cell infusion has been proven to be critical for the anti-tumor efficacy of CAR T cells and has been demonstrated in many murine CAR T cell models. In a murine model of lymphoma, a mouse CD19-targeting CAR (1D3-28Z.1-3) was shown to only be effective if the mice received irradiation preconditioning prior to CAR T cell administration [30]. This was further confirmed in a leukemia model using the same CAR T cells that demonstrated that either irradiation or cyclophosphamide preconditioning was needed for effective CAR T cell activity [31].

Natural killer group 2-member D (NKG2D) is a receptor expressed on NK cells that binds to a plethora of ligands expressed on tumor cells. The binding capabilities of this receptor have been utilized as the binding domain for several human CAR T cells [32,33]. A first-generation murine NKG2D CAR (chNKG2D) was created by fusing the murine CD3ζ chain cytoplasmic region to full-length murine NKG2D. chNKG2D T cells significantly extend the survival of mice in a syngeneic orthotopic glioma model compared to T cells expressing NKG2D without the CD3ζ domain (wtNKG2D) [33]. In this model, the use of cranial irradiation increased the efficacy of the CAR, improving survival and decreasing tumor volume relative to the lone CAR T cell group. When further investigated, it was shown that the irradiation of tumors caused increased IFNγ release by CAR T cells and improved CAR T cell trafficking to the tumor [34].

In an immunocompetent leukemia model, it was shown that murine CD19 CAR T cell therapy was ineffective without prior lymphodepletion via cyclophosphamide or irradiation [31]. Similarly, another group found that CD19-targeted CAR T cells could eliminate established lymphoma in syngeneic models. However, optimal effectiveness was dependent on the successful engraftment of gene-modified T cells, which required administering CAR T cells following cyclophosphamide lymphodepletion [35]. Optimization of a preconditioning regime was performed in a syngeneic model of B-cell acute lymphoblastic leukemia (B-ALL). In this study, murine CD19-targeting CARs were combined with varying amounts of preconditioning chemotherapy. The study showed that increasing the dose of cyclophosphamide improved tumor eradication and also improved CAR T cell persistence within the mice [36].

Separate studies established a third-generation EGFRvIII-specific mCAR. They targeted a murine homologue of EGFRvIII that has similar antibody-binding features to the human EGFRvIII [37]. The murine 139 scFv mCAR was made similarly to the human (h) 139 scFv hCAR and inserted in tandem to the mCD8 transmembrane, mCD28, m4-1BB and mCD3ζ intracellular regions. The transduced T cells expressed interferon-gamma (IFNγ) in the presence of EGFRvIII mutated target cells. The EGFRvIII mCAR T cells, infused into VM/Dk mice bearing SMA560vIII gliomas at high doses (7 × 10^6^ mCAR T cells/mouse), were fully curative for all mice with CNS tumors. However, they were all dependent on lymphodepletion and host conditioning [37]. The lymphodepletion-dependent anti-tumor efficacy seen in this study was speculated to be due to a reduction in immunosuppressive cells and cytokines allowing the CAR T cells to expand. Furthermore, these cured mice were resistant to rechallenge with EGFRvIII^NEG^ tumors, indicating the development of immunity against additional tumor antigens [37].

CAR T cells targeting CD70, a novel target in gliomas, have also been investigated in immunocompetent models. CD70-targeting CAR T cells were generated using its ligand, full-length murine CD27, fused with murine CD3ζ (pMSGV8-mCAR) [38]. In a syngeneic model of glioma, treatment with two doses of 1 × 10^7^ pMSGV8-mCAR T cells resulted in improved survival compared to untransduced T cells or untreated mice. Complete tumor regression was also seen in some mice. Additionally, a human CD70-targeting CAR T construct was also produced using the human CD27 as the binding domain and a 4-1BB costimulatory domain. This construct also demonstrated complete tumor regression in NSG mice treated with 10^7^ CAR T cells. No negative adverse effects were detected in the syngeneic or xenograft models treated, establishing CD70 CAR safety in vivo and their potential for treating patients with refractory gliomas [38]. In this model, full-body (FB) irradiation not only caused upregulation of the target antigen, but also increased CAR T cell IFNγ release in response to antigens, illustrating the importance of preconditioning on CAR T cell efficacy [38].

## 5. Predicting Toxicities

### 5.1. Cytokine Release Syndrome

Cytokine release syndrome (CRS), characterized by respiratory distress, fever and hypotension, is a major toxicity seen in patients treated with CAR T cells [39]. Recently, it has been shown that CRS is mediated by cytokines (particularly IL-1 and IL-6) not released by the CAR T cells themselves but by other immune cells, such as macrophages and monocytes, in response to the CAR T cell therapy [40]. Cytokine release syndrome (CRS) cannot be fully recapitulated in immunocompromised mice as the host immune system plays a crucial role in the development of this toxicity. This is a major barrier to developing effective and safe CAR T cell therapies.

### 5.2. On-Target–Off-Tumor Toxicity

The importance of pre-clinical testing of CAR T cells in a suitable mouse model was demonstrated in the first human trial of an anti-HER2 CAR T cell [41]. The pre-clinical testing in immunodeficient xenograft models was unable to anticipate dangerous toxicities caused by on-target–off-tumor CAR activity in the lungs. Sometimes, the target patients enrolled in a trial of CEA-specific T cells developed autoimmune colitis that required halting the therapy [42].

Siegler and Wang et al. discussed that when there is no human homolog in a mouse, this compromises the ability to establish an accurate safety profile; for example, there is no mouse equivalent of CEACAM7, which was recently identified as a potential target in pancreatic cancer [43]. Similarly, on-target–off-tumor toxicities should be studied in syngeneic mice as, in most cases, human CAR T cells do not recognize the homologous mouse antigens that may be expressed in the healthy tissue [44]. To add to this, Yaguchi et al. showed that the anti-tumor response and efficacy of combination immunotherapy cannot be studied effectively in xenogeneic models due to the incompetence of the immunodeficient host immunity to respond fully to the tumor, which can lead to alterations in the CAR T cell phenotype [45].

The use of fully syngeneic models can lead to clinical trials in humans. An example of this is the natural killer group 2-member D (NKG2D)-based CAR that was initially tested in an immunocompetent mouse model of glioblastoma. This full-length murine NKG2D protein was fused with a CD3ζ and DAP10 costimulatory domain to produce a second-generation CAR (chNKG2D) [46,47]. A control construct was also made just with the NKG2D domain but no intracellular CD3ζ domain (wtNKG2D). These CAR T cells were injected into syngeneic C57BL/6 or VM/Dk (a spontaneous glioma model) mice. The murine chNKG2D CAR T cells in combination with irradiation could produce elevated IFNγ levels and increase cytolytic activity in vivo. They were capable of migrating to the tumor site, increasing survival, and even cured a fraction of glioma-bearing mice [34]. Although targeting the NKG2D ligand had the potential to induce on-target–off-tumor toxicity, no side effects were observed in mice exhibiting stable body weight, normal liver function and peripheral blood counts following administration of the CARs. Following these studies, a phase-one human trial (NCT02203825) assessing the NKG2D CAR T cells against hematological malignancies (acute myeloid leukemia/MDS/multiple myeloma) completed enrolment of a cohort of patients with no treatment safety issues [34].

This human study also reported no cases of cytokine release syndrome, neurotoxicity, auto-immune reactions or CAR T induced death. However, SAEs (severe adverse effects), including grade 4 intracochlear bleeding, neutropenia, thrombocytopenia and grade 3 toxicity, were noted. With the initial dosing, no patient had an objective tumor response during day 28 of the evaluation; while nine initiated subsequent therapies, four died secondary to disease or complications. However, an unexpected survival case was noticed in a p53-positive AML patient that survived 4 months even with a high disease burden [48].

The most successful CAR T cell target to date is CD19, expressed on B cell malignancies. The anti-tumor effect of these CAR T cells was demonstrated originally with human CAR T cells in vitro and in immunodeficient mice [49].

However, these models could not account for the CD19 CAR T cell-targeting of normal B cells, which also express CD19, and thus it was necessary to evaluate the safety of this on-target–off-tumor activity. To accomplish this, a murine CD19-targeting CAR (1D3-28Z.1-3) was used in a syngeneic model of lymphoma. This study demonstrated complete and long-term tumor eradication in addition to normal B cells. B cells were still not detectable in the spleen of these mice 143 days after 1D3-28Z. Afterwards, 1–3-transduced T cells were administered, showing the long-term effects of the on-target–off-tumor activity of the CARs. B cell aplasia is also seen in humans treated with CD19 CAR T cells but can be treated by the administration of intravenous immunoglobulin (IVIG) replacement therapy [30].

GD2 has been identified as a promising target for CAR T therapy on certain neuronal tumors. An early study that targeted GD2 in relapsing neuroblastoma showed favorable results in phase 1 trials, even though they used first-generation CARs, which are less efficacious than the latest constructions [50]. The CAR construct they used was generated with human Ig signal peptides, the scFv from 14g2a, a GD2-specific monoclonal antibody, the hinge region of IgG1 and the transmembrane and intracellular portions of CD3ζ [51,52]. To see if they could improve CAR T cell function, another group introduced modifications to the hinge and the scFv affinity of the human GD2 CAR. The modifications resulted in enhanced CAR T cell function in vitro and in vivo compared to the original CAR structure in immunodeficient models. However, these modifications resulted in lethal on-target–off-tumor neurotoxicity. These data provided key risk information to those targeting GD2 [50]. To further investigate this question, a murine CAR construct was constructed using the 14G2a murine antibody-based scFv, a CD8α hinge and transmembrane domain, a 4-1BB costimulatory domain and a murine CD3ζ signaling domain (E101K-CD28-CD3ζ CAR) [50]. All mice treated with the E101K-CD28-CD3ζ CAR developed severe neurotoxicity by day 9 after treatment. This model also brought to light some of the mechanisms of T cell trafficking into the CNS, which is usually restricted by the blood–brain barrier. While GD2-positive tumors are rarely CNS tumors, selective inhibition of the trafficking T cells could potentially decrease the risk of CNS toxicity when treating other GD2-positive cancers such as multiple myeloma and metastatic melanoma [50].

CXCR5 has also been investigated as a target for B cell non-Hodgkin’s lymphoma (B-NHL). CXCR5 is a chemokine receptor expressed on mature B cells, malignant B cells and T follicular helper cells (Tfh). CXCR5 is involved in homing and trafficking lymphoid cells to B cell follicles in secondary lymphoid organs [53]. A human CXCR5-targeting CAR T cell showed in vitro efficacy against a range of cell lines and patient-derived primary tumor cells, and tumor eradication in an NSG model of B-NHL. However, during relapse in this model, they noted persistent CXCR5 antigen expression on the tumor cells, which meant that murine cytokines did not effectively support the long-term survival of human anti-CXCR5 CAR T cells. To further investigate this, an anti-murine CXCR5 CAR construct was generated and tested in a murine model of lymphoma, which demonstrated anti-tumor efficacy as well as normal B cell depletion. Additionally, the CAR T cells could target CXCR5 expressed on T follicular helper cells (Tfh) cells in the lymphoma niche [54]. Tfh cells play an important role in tumor cell proliferation and resistance to therapy, so targeting them with CAR T cell therapy could provide additional anti-tumor effects. Finally, they also found that there was no reactivity against CXCR5-negative precursor B cells, demonstrating the safety of this approach. The conserved expression patterns of CXCR5 in the B cell lineage in mice and humans allowed the murine model to be used to test the safety of this approach [55].

Modern cancer therapy acknowledges that the cells and proteins in the tumor microenvironment contribute significantly to tumor progression. Stromal cells such as cancer-associated fibroblasts (CAFs) in the TME contribute to tumor growth and therapeutic resistance by forming a physical barrier, providing support for invasion and angiogenesis, secreting immunosuppressive factors that regulate T cells and alter myeloid phenotypes, and expressing immuno-inhibitory surface molecules [56]. To test this hypothesis, a transmembrane serine protease target, the fibroblast activating protein (FAP), was selected due to its increased expression on cancer-associated stromal cells in many epithelial tumors [57,58]. The anti-FAP CAR construct contained a murine CD28 costimulatory domain and a murine CD3ζ activation domain. Murine CAR T cells targeting FAP were used to treat subcutaneous tumors (mesothelioma or lung cancer) growing in either immunodeficient or immunocompetent mice. Interestingly, it was found that the tumors grew faster in the NSG mice and that there was no difference in tumor size or survival seen between the untreated and FAP CAR-treated groups in these mice. Contrastingly, the C57BL/6 mice showed a significant reduction in tumor volume in mice treated with the FAP CAR, and this also correlated with an increased number of host CD8+ T cells within the tumor and higher numbers of TNF-α-producing CD4^+^ T cells [59]. Thus, the syngeneic experiments suggested that the CAFs were contributing to immunosuppression and that the anti-FAP CAR T cells eliminated this, restoring the host’s immune response. These observations were not revealed in the immunodeficient model.

Building on these findings, the authors combined their FAP-targeting CAR T cells with an adenovirus expressing HPV-E7. The vaccine was administered to enhance the endogenous immune response to E7, a tumor vaccine to enhance the endogenous T cell response against tumor cells expressing E7 [59]. Single-agent therapy with vaccine or murine FAP-targeting CAR T cells had little effect on large E7-expressing subcutaneous lung cancer tumors, but the combination of these two treatments resulted in delayed tumor progression and significantly smaller tumor volume on day 21 post-tumor cell inoculation. Such combination studies can only be carried out in a syngeneic model due to the reliance on the endogenous immune cells for activity [59].

### 5.3. Graft-Versus-Host Disease (GvHD)

Immunodeficient models have an increased risk of developing graft-versus-host disease (GvHD), which occurs when the administered human T cells recognize the host tissue as foreign [23]. GvHD in mice can lead to weight loss, ruffled fur and fatal immune responses [60]. One study investigating glypican-1 (GPC1)-targeting CAR T cells demonstrated that the anti-tumor effects observed in immunodeficient mice may be hampered by the development of GvHD. They found that when they tested the anti-tumor effects of human CAR T cells against GPC1 in xenograft solid tumor models, the rate of tumor eradication was slower than in immunocompetent models using murine GPC1-targeting CAR T cells. This study suggested that GvHD in the xenograft model resulted in the late activation and expansion of human CAR T cells when compared to syngeneic mouse models, resulting in slower tumor clearance [61].

In the clinical setting, most CAR T cells are produced from the patient’s own T cells, and therefore do not pose a risk of GvHD. However, if a patient has received a bone marrow transplant (BMT) as part of their treatment, it may be possible to manufacture the CAR T cells from the BMT donor [62]. This can be beneficial as the T cells of cancer patients are often dysfunctional [63]. To model this phenomenon, murine anti-CD19 CAR T cells were utilized in an immunocompetent acute lymphoblastic leukemia (ALL) model. Mice received donor minor-mismatched CAR T cells following a BMT, which were able to cause the remission of residual CD19+ leukemic cells. However, these mice developed lethal GvHD. This was not seen in mice receiving autologous CAR T cells. GvHD was only induced in mice that had CD19+ leukemia cells present, and not in those that received only allogeneic CAR T cells with no tumor burden. The authors found that IL-6, produced by the allogeneic CD4+ CAR T cells, was partially responsible for the GvHD. IL-6 is a pro-inflammatory cytokine that has been implicated in chronic inflammation resulting in tissue damage [64]. Blocking IL-6 may be clinically valuable to reduce GvHD in these patients. These data demonstrate further the importance of syngeneic models for the pre-clinical evaluation of CAR T cells [31].

### 5.4. Recapitulating Immunosuppression

The solid tumor microenvironment TME is flooded with multiple immunosuppressive cells and soluble factors that act to suppress effector cell function. One of the predominant cells involved in immunosuppression is regulatory T cells (Treg). These cells act to suppress cytotoxic effectors, including CAR T cell effector function, via direct cell-to-cell interactions, as well as by the release of immunosuppressive soluble factors. For example, Tregs express high levels of fas ligand on their surface, which can induce fas-mediated apoptosis in CAR T cells [65,66]. Tregs also use granzyme B and perforin to induce effector T cell apoptosis, and galectin-1 released by Tregs is able to induce apoptosis in activated T cells [67,68]. Tregs also produce several immunosuppressive cytokines, including TGF-β, IL-10 and IL-35. Broadly, these cytokines suppress effector T cell proliferation [69,70]. TGF-b can also directly inhibit the cytotoxic function of T cells [71,72]. In addition, Tregs express high amounts of CD25, which binds to IL-2. This sequesters the IL-2 in the TME and prevents it from being utilized by the CAR T cells [73,74].

M2 tumor-associated macrophages (TAMs) are another important immunosuppressive cell present in the TME. They express PD-L1, which can induce anergy in effector T cells [75]. Additionally TAMs produce prostaglandin E2 (PGE2), which inhibits IFNγ production in effector T cells [76]. Myeloid-derived suppressor cells (MDSCs) express Indoleamine 2,3-dioxygenase (IDO), an enzyme involved in tryptophan metabolism [77,78]. The metabolites produced cause reduced proliferation and cytotoxicity in CAR T cells [27]. IDO is also expressed in many tumor cells, further increasing the immunosuppression in the TME [79]. Thus, whilst xenograft models evaluate the efficacy of CAR T cell treatment, they fail to provide any information about the response of an immunocompetent TME, which affects CAR T cell efficacy [80].

The inhibitory effect of the host immune system was demonstrated in a study investigating liver myeloid-derived suppressor cells in colorectal cancer liver metastasis. The authors used a second-generation CAR made of a hMN14 sFv-CD8α fused to human CD28 and CD3ζ CARs [81]. In mice, MDSCs are characterized by the CD11b + Gr-1+ phenotype [82]. When CEA CAR T cells were co-cultured with CEA+ target cells, the presence of L-MDSCs suppressed CAR T proliferation two-fold. However, this effect could be reversed by blocking PD-L1 using an antibody or by making the CAR T cells from mice that lack the ligand (PD-1−/− mice) [83]. To investigate if the depletion of L-MDSCs in vivo can improve CAR T cell efficacy, an anti-Gr-1 antibody was administered in combination with anti-CEA CAR T cells or untransduced T cells. Anti-CEA CAR T with L-MDSC depletion resulted in significantly fewer viable liver metastasis cells compared with either monotherapy or anti-Gr-1 antibodies with untransduced T cells. The anti-CEA CAR T and anti-Gr-1 antibody groups also had significantly prolonged survival [83]. Unfortunately, in humans, MDSCs cannot be classified using CD11b + Gr-1+, so an anti-Gr-1 therapy cannot be used. The authors therefore investigated using GM-CSF neutralization as a clinically translatable alternative for MDSC depletion. GM-CSF is released by tumor cells and causes MDSC recruitment [84]. They therefore assessed the in vivo efficacy of anti-CEA CAR T cells in combination with anti-Gr-1, anti-GM-CSF, anti-PD-L1 or the combination of anti-GM-CSF and anti-PD-L1. The authors saw a significant increase in the survival of mice treated with CAR T + anti-GM-CSF compared to CAR T alone (*p* = 0.03) [83]. Following on from this work, a trial was performed using anti-CEA CAR T infused regionally into patients with unresectable CEA+ liver metastasis (NCT01373047).

Another approach being investigated is targeting the immunosuppressive cells in the TME using CAR T cells. Folate receptor β (FRβ) is a glycophosphatidylinositol-anchored receptor expressed on the TAMs in many tumor types [85]. FRβ expression is restricted to immunosuppressive M2-like macrophages [86]. In vitro, the proliferation of anti-human mesothelin (hMeso) mouse CAR T cells was significantly reduced in the presence of FRβ^+^ TAMs as compared to FRβ^−^ TAMs, and this was accompanied by a reduction in IFN-γ production by the CAR T cells [86]. In in vivo models of melanoma, ovarian and colon cancer treatment with mFRβ-targeting CAR T cells led to the specific depletion of the FRβ^+^ TAM population compared to control hCD19 CAR or untransduced T cells 6 days after T cell infusion. The CAR-mediated depletion of FRβ^+^ TAMs resulted in delayed tumor growth and improved survival compared to hCD19 CAR or untransduced T cell-treated groups, despite the lack of mFRβ on the tumor cells [86]. Mice treated with the mFRβ CAR T cells had a more pro-inflammatory TME (including inflammatory monocytes and neutrophils) compared to hCD19 CAR or untransduced T cell-treated groups, demonstrating the CAR T cell-mediated remodeling of the TME. They also had more CD8 T cells and these had a more activated phenotype. When this experiment was repeated in a CD8 KO mouse, there was no tumor reduction, illustrating that the endogenous CD8 T cells are the likely mediators of the anti-tumor effect [86]. When the authors administered the mFRβ CAR T cells prior to murine hMeso CAR T cells, it resulted in improved survival and tumor reduction compared to monotherapy. It also resulted in more persistence of the hMeso CAR T cells [86]. This study illustrates that modifying the TME, even with another CAR, can improve CAR T cell efficacy.

## 6. Modeling Beneficial Interactions with the Host Immune System

A recent phase 1 clinical trial suggested that in patients with gliomas, the endogenous immune system positively affected therapeutic outcome of CAR adoptive therapy. In light of this, testing with a syngeneic mouse model in pre-clinical studies would feasibly have helped generate more accurately the likelihood of a positive therapeutic outcome [87]. These reports further underline the need to assess the safety profile and antigen expression of adoptive CAR T cells in syngeneic models where antigen expression profiles closely match human distribution [88].

**Figure 1:**
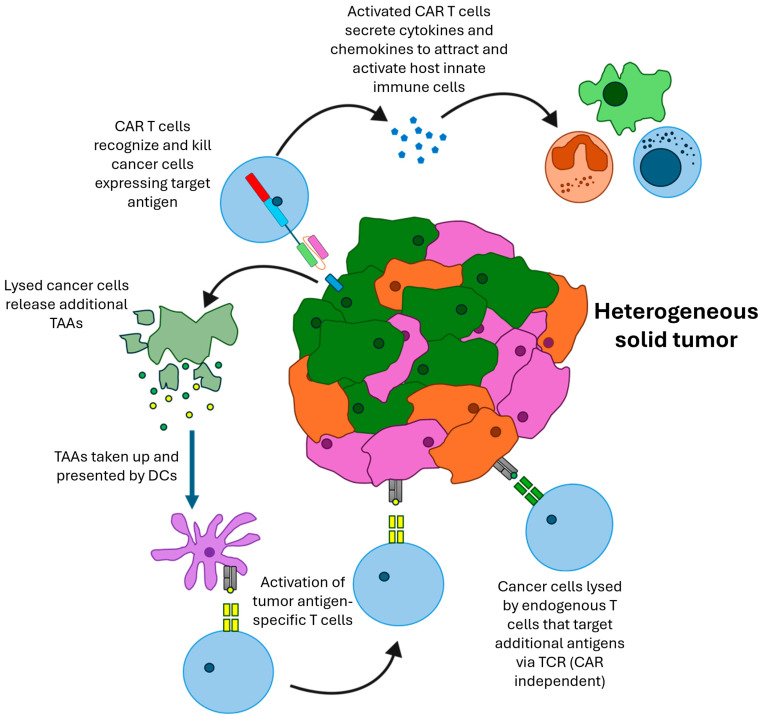
CAR T cell bystander effect in heterogenous solid tumor. CAR T cells target cancer cells expressing target antigens. Activation of CAR T cells causes secretion of cytokines and chemokines that can recruit and activate neutrophils, macrophages and NK cells. CAR T cells directly lyse cancer cells, which release tumor associated antigens (TAAs). These TAAs are taken up by dendritic cells, which can present them to endogenous T cells. Native T cell receptors (TCRs) specific for these TAAs are activated and move to the tumor site. These T cells can target TAAs that are not targeted by the CAR, resulting in CAR-independent cancer cell lysis.

One major obstacle to successful CAR T cell therapy in solid tumors is the heterogenous expression of tumor antigens. For this reason, it is unlikely that the CAR T cells will be able to target every cancer cell within the tumor. However, the “bystander effect” caused by the host immune system’s destruction of CAR-target antigen-negative tumor cells following CAR T cell cytotoxic activity has been noted (Figure 1) [89]. The mechanisms are not fully understood but one component is thought to be epitope-spreading, whereby tumor-associated antigens (TAAs) are released upon the CAR T cell-mediated lysis of tumor cells. These antigens (different to the CAR-targeting antigen) are taken up by antigen-presenting cells (APCs) such as macrophages and dendritic cells and can induce a B and T cell response against these other TAAs [90,91,92]. Additionally, the activity of the CAR within the tumor can release cytokines such as IL-2, which can help activate and support the host immune cells. Murine models that lack functional immune systems cannot fully reflect the mechanisms of tumor eradication because it is not possible to prime and enhance the native immune cells, which would result in responses that modify anti-cancer therapies.

To test the bystander hypothesis, murine mesothelin-targeting (mesoCAR) CAR T cells were used against tumors in immunocompetent mice. These CAR T cells could eradicate tumors that were 100% antigen-positive; however, the mesoCAR T cells were unable to cure tumors even when only 10% of tumor cells were mesothelin-negative, even with the co-administration of (1) immune modulatory agents such as anti-PD-1, anti-CTLA-4 or anti-TGF-β (transforming growth factor β) antibodies; (2) agonistic CD40 antibodies; or (3) an IDO (indoleamine 2,3-dioxygenase) [89]. Interestingly, when low-dose cyclophosphamide was used to pretreat this mouse model, tumors were cured even when they contained up to 25% mesothelin-negative cells (without co-administration of any other immune modulating agents). To investigate the mechanism behind this, the authors compared the effect in BATF3 knock-out (KO) C57BL/6J and wild-type mice. BATF3 KO mice lack conventional dendritic type 1 (DC1) cells, which are responsible for antigen presentation within tumors [93]. In these knock-out mice, cyclophosphamide in combination with mesothelin CAR T cells was able to cure mice with tumors containing 10% mesothelin-negative cells. However, in a CD8 knock-out model, mice were not cured when receiving the same treatment. This illustrates that the bystander effect in this model is mediated by endogenous CD8 T cells but the DC1 cells were not required to educate them. This study emphasizes that adoptive therapy for solid tumors with antigen targets not widely expressed on tumor cells will be successful if (1) a sufficient fraction of the tumor cells express the target antigen and (2) additional strategies are used to increase natural host immunity to generate effective bystander effects [89]. Interestingly, IL-12-expressing murine CD19-targeting CAR T cells have shown promising results in established lymphomas in part because the IL-12-induced epitope-spreading of the tumor-associated antigens without a requirement for lymphodepletive preconditioning [94].

The interaction between myeloid and CAR T cells was investigated by Spear et al. [95]. They used a first-generation murine NKG2D CAR (chNKG2D) in an immunocompetent ovarian cancer model. They found that CAR T cells recruited peripheral myeloid cells to the TME in a CCR2-dependent manner. They repeated this experiment using CAR T cells made from GM-CSF- or IFN- γ-deficient mice. The authors found that the increase in peripheral myeloid cell recruitment was maintained in the IFN-γ-deficient CAR T cells but was lost in the GM-CSF-deficient CAR T cells, illustrating that the CAR T cell-mediated recruitment of myeloid cells is GM-CSF-dependent [95]. When the host macrophages were depleted using clodronate liposomes in combination with chNKG2D CAR T cells, they saw an increase in the number of solid tumors on the peritoneal wall compared to chNKG2D CAR T cells alone, highlighting the important role of host macrophages in CAR T cell-mediated tumor clearance [95]. This study showed that cytokines released from CAR T cells are vital for the activation of host macrophages, which can transform the tumor TME from an immunosuppressive to immunostimulatory environment, and illustrates the importance of the host immune system in tumor clearance by CAR T therapy. Such conclusions could not have been achieved using immunodeficient mouse models.

## 7. Combination Therapies

As seen by the lack of clinically successful CAR T cell therapies in solid tumors, it is evident that combination therapies may be necessary to realize the full potential of CAR T cells. The modulation of the host immune system is an attractive target for combination therapies; however, these treatment regimes require pre-clinical testing in immunocompetent models. In one study IL-18-secreting murine CAR T cells were tested in immunocompetent syngeneic models [96]. IL-18 is released by macrophages and induces IFNγ expression in T cells, making it an attractive cytokine to use in combination with CAR T cells [97]. In the study, murine T cells were modified with a number of CAR retroviral vectors targeting CD19: 19m28mz—a second-generation CD19 CAR; 19m28mz-mIL18—a second-generation CD19 CAR that secretes mIL-18; and two constructs that lack intracellular signaling domains as controls (19mDel and 19mDel-mIL18). T cells expressing 19m28mz-mIL18 produced over five times more IL-18 (*p* = 0.004) and showed increased proliferation (*p* = 0.003) in vitro in the presence of the thymoma cell line EL4 (modified to express CD19 (EL4hCD19+)) compared with 19m28mz CAR T cells. The 19m28mz-mIL18 CAR T cells were further tested against the aggressive syngeneic tumor model EL4hCD19+ to observe whether they could surpass the efficacy of the second-generation 19m28mz CAR T cells. The results showed that these 19m28mz-mIL18 CAR T cells (2.5 × 10^6^ CAR cells/mouse), in the absence of preconditioning, could successfully and significantly (*p* < 0.001) enhance the long-term survival of the EL4hCD19+-bearing syngeneic mice when compared to mice subjected to 19m28mz CAR T cell treatment. Anti-tumor efficacy was also achieved even when EL4hCD19+-bearing mice were treated with a half CAR T cell dose (1.2 × 10^6^ CAR cells/mouse) of the 19m28mz-mIL18 CAR T cells, and the mice also rejected a second lethal tumor dose, hence retaining their efficacy on tumor rechallenge. In order to establish whether the 19m28mz-mIL18 CAR T cells had the ability to migrate to the tumor site and elicit their IL18-dependent effects on the endogenous immune system, they were injected into immunocompetent syngeneic mice bearing EL4hCD19+ tumors, and at time points thereafter, tumor tissue was analyzed by imaging mass cytometry (CyTOF). The CyTOF results confirmed successful migration of 19m28mz-mIL18 CAR T cells to the bone marrow (compared to controls) and detection of these CAR T cells at least 18 days after infusion. Interestingly, these 19m28mz-mIL18 CAR T cells were capable of activating the endogenous immunity of the bone marrow against EL4hCD19+ cells, and those mice showed increased CD8 T cells with a central memory phenotype, macrophages of M1 phenotype and mature dendritic cells with an activated phenotype. The authors found that the targeted delivery of IL-18 modulated the EL4CD19 TME and activated endogenous antineoplastic responses, which allowed the lysis of target antigen-negative cells [96].

Another cytokine of interest is IL-12, a pro-inflammatory cytokine that has a plethora of anti-tumor effects. IL-12 can boost the cytotoxicity of CAR T cells, improve antigen presentation, decrease the activity of regulatory T cells (Tregs) and activate myeloid cells while resulting in negligible systemic effects [98,99,100]. The beneficial effect of IL-12 with CAR T cells is evident in a study using an immunocompetent murine GBM model. This study showed that EGFRvIII CAR T cells alone failed to control established tumors, but when the CAR T cells were combined with a single dose of intra-tumoral recombinant-chain IL-12, they caused a significant anti-tumor response. This combination successfully overcame the failure of CAR therapy in previous GBM studies [98]. IL-10 is another cytokine that has been investigated to improve the function of CAR T cells. It has been shown that IL-10 can cause metabolic reprogramming in terminally exhausted CD8+ T cells [101]. Armoring murine CAR T cells with IL-10 reduced T cell dysfunction and enhanced tumor clearance in syngeneic models of colon cancer and melanoma. Armoring the CAR T cells with IL-10 enhanced proliferation, induced a stem cell-like memory (Tscm cell) phenotype and increased mitochondrial activity in the T cells [102].

Similarly, murine CAR T cells targeting GD2 or B7-H3 were modified to undergo autocrine IL-23 signaling. This was achieved by expressing the p40 subunit of the IL-23 in addition to the CAR, resulting in IL-23 production when the T cells were activated [103]. This system was tested in two immunocompetent CAR T cell models, GD2 in a neuroblastoma tumor and B7-H3 in a PDAC tumor. Both CAR T cells modified to express the mouse p40 subunit showed enhanced tumor control and prolonged survival compared to the CAR T cells alone [103]. This antineoplastic activity correlated with an elevated granzyme B release and a decrease in PD-1 expression by the CAR T cells. In these models, IL-23 expression was restricted to the TME, as it was only released upon CAR T cell activation. This reduced potential systemic effects from the cytokine [103]. Likewise, co-expression of murine interleukin-15 with a second-generation murine CAR targeting vascular endothelial cell growth factor receptor 2 (VEGFR-2) showed enhanced tumor control in a syngeneic melanoma model compared to CAR alone. The mIL-15 CAR T cells also showed significantly lower upregulation of PD-1 compared to the CAR T cell alone [104].

Another major obstacle that reduces the efficiency of adoptive cell therapies is the inhibitory action of PD1/PDL1 signaling by the tumor cells. In an attempt to maximize the efficacy of CAR T cells, several studies have explored the use of monoclonal antibodies targeting this axis. This was demonstrated using a third-generation anti-HER2 mCAR containing CD28 and CD137 costimulatory domains. In this study, CAR T cells were combined with an anti-PD1 antibody to target HER 2-expressing breast tumor cells in vivo in BALB/C mice [80]. In vitro, at a 16:1 effector:target ratio, the percent cytotoxicity for anti-HER2 CAR T cells alone was 39.8%; however, the addition of PD1 antibodies increased cytotoxicity to 49.5% (*p* < 0.001). The addition of the PD1 antibody also increased IL-2 and IFN-γ secretion. CAR T cells cultured with anti-PD1 antibodies also displayed enhanced expansion compared to CAR T cells alone in the presence of HER2+ target cells. In vivo combination therapy also showed favorable results, demonstrated by a 73.3% decrease in tumor weight in the CAR T plus anti-PD1 group, compared to a 50% decrease in CAR T cells alone. Tumors treated with the anti-PD1 antibody also reported the highest fraction of apoptotic tumor cells (63.28%) amongst the treatment cohorts. The T cell infiltration into the tumors was significantly higher in the CAR T cell-treated groups than in the untransduced T cell treatment group, but the greatest T cell infiltration was seen when CAR T cells were combined with the anti-PD1 antibody group [80]. In a similar fashion, a CAR T cell directed against either CD19 or MUC16^ecto^, modified to secrete a PD-1 blocking scFv, was shown to increase the survival of mice with PD-L1^+^ tumors in both syngeneic and xenogeneic models, and was effective in confirming the delivery of immune-modulatory scFvs by CAR T cells in the TME [105].

Another aspect that has been explored in immunocompetent models is genetic knock-outs of specific genes in the CAR T cells. These modifications can improve the persistence of the CAR T cells and make them more resistant to dysfunction in vivo. As discussed above, the PD-1 axis is a key player in the suppression of T cells in the TME. To reduce the potential toxicities of checkpoint inhibitors, the genetic ablation of PD-1 in CAR T cells has been investigated. This has been previously investigated in immunodeficient models and in clinical trials [106,107,108,109]. Dötsch et al. investigated the effect of PD-1 KO in an immunocompetent model of ALL [110]. In these experiments, Cas9 ribonucleoprotein (RNP) gene-editing was used to make PD-1 KO T cells that were also retrovirally transduced with murine anti-CD19 CARs. These cells were used to treat ALL in C57BL/6 mice. The PD-1 KO CAR T cells showed stronger expansion in vivo than the CAR T cells alone, and showed equivalent persistence within the mice, up to 390 days [110]. Similarly, another study showed that CRISPR-Cas9-mediated inhibition of the E3 ubiquitin ligase Cbl-b, shown to be upregulated in exhausted CD8+ TIL, improved the function of CEA-targeting CAR T cells. Cbl-b–/–anti-CEA CAR T cells improved the survival of Rag–/– mice bearing CEA+ MC38 tumors compared to Cbl-b+/+ CAR T cells (*p* = 0.002). The Cbl-b–/– CAR T cells also reduced tumor volume in these mice compared to Cbl-b+/+ CAR T cells (*p* = 0.000008) [111].

Other combination therapy approaches include combining adoptive T cell transfer with oncolytic adenoviruses (OAds) that express immunomodulatory transgenes. One study utilized an adenovirus expressing murine TNF-α and IL-2 (Ad-mTNFa-mIL2) in combination with mouse mesothelin-targeting (mmeso) CAR T cells. In an orthotopic pancreatic cancer model, mmeso CAR T cells failed to suppress tumor growth; however, combining the CAR T cells with Ad-mTNFa-mIL2 was reported to increase CAR T cell and host T cell infiltration, as well as M1 polarization of macrophages [112]. These combination therapies can only be tested in immunocompetent models as their efficacy requires a functioning host immune system [112].

Another study investigated the combination of an oncolytic vesicular stomatitis virus (VSV) encoding mouse interferon-β (mIFNβ) with a third-generation anti-epidermal growth factor receptor variant III (EGFRvIII) CAR T cell. It was hypothesized that the virus would enhance the recruitment and function of the CAR T cells by inducing inflammation within the tumor [113]. In a syngeneic model of melanoma, VSVmIFNβ was administered intra-tumorally 6 h prior to CAR T cell infusion. Single-agent treatments resulted in a reduction in tumor growth, but there was no significant benefit seen in the combination group. Additionally, the authors observed a 3-fold reduction in CD8 CAR T cells detectable in the blood 7 days after transfer in the combination group compared with the CAR T cell-only group. Similar results were seen when a preconditioning treatment was given prior to CAR T cell administration. To investigate the mechanism behind this, the authors used splenocytes from transgenic interferon receptor alpha (IFNAR) KO (CD45.2) mice to make the CAR T cells. These cells, lacking the IFNAR, had a survival advantage in the combination treatment compared to WT mice. They found that cells exposed to IFNβ underwent enhanced activation and were more sensitive to apoptosis. They then showed that using T cells with a CRISPR Cas9-mediated disruption of IFNAR1 could result in improved survival and reduced tumor growth in mice treated with VSVmIFNβ and CAR T cells compared to CAR T cells alone [114].

These authors then investigated administering the virus 5 days after CAR T cells. Using this schedule, they found no CAR T cell attrition. Using this regime, they detected an enhanced expansion of CD8 CAR T cells that had a native TCR against the immunodominant epitope of the virus, named dual-specific (DS) CAR T cells. They found that DS CAR T cells made up 25% of the CAR T cell population in vivo and when isolated, these cells displayed enhanced cytokine production and degranulation against EGFRvIII-expressing cells. To expand this concept further, they preloaded the CAR T cells with a virus, thereby ensuring co-delivery of both the virus and the CAR T cell. They found that virus-loaded CAR T cells improved the survival of mice with subcutaneous melanoma compared to mice receiving unloaded CAR T cells (*p* = 0.003) [115]. Evgin and colleagues then proposed that this method could help target CAR antigen-negative cells. To test this, they treated mice bearing brain stem tumors with 100% antigen-positive cells or a mixed population with only 10% antigen-positive cells. Virus-loaded CAR T cells improved survival compared to individual or combined treatment with the virus or CAR T cells, but did not result in tumor cure. However, a dose of virus-loaded CAR T cells followed by a further systemic dose of the virus further improved efficacy and resulted in tumor cures in 2/7 mice [115]. This study illustrates the benefit of combination therapy, particularly its ability to induce epitope spreading.

With the use of standard CAR therapy, developing patient-specific CAR T cells with a particular antigen specificity is time-consuming and labor intensive. Hence, one study reported that using anti-FITC CAR T cells would allow therapy directed against multiple tumor targets, provided they were pre-treated with FITC-labeled anti-tumor antibodies [116]. Anti-FITC mouse CAR T cells generated from murine splenic T cells exhibited potent cytotoxic activity against tumors in immunocompetent syngeneic models.

Immunocompetent mice were inoculated with human CD20-expressing 38C13 mouse tumor cells (as a rituximab-insensitive B-cell lymphoma model) and treated with anti-FITC CAR T cells combined with either FITC-Rituximab or non-fluorochrome-conjugated Rituximab. Mouse survival in those treated with FITC-Rituximab was significantly longer than in those with non-labeled Rituximab (*p* < 0.001) [116]. The authors proceeded to measure the therapeutic effect of anti-FITC CAR T cells in the mouse Her2-expressing breast cancer model E0771/E2 by transferring syngeneic C57BL/6-derived mouse anti-FITC CAR T cells combined with FITC-labeled trastuzumab and noted a rejection of the pre-established E0771/E2 tumor cells that was not seen in the Her2-negative parental tumor [116]. This result reflects the positive ability of anti-FITC CAR T cells to stimulate a therapeutic effect against syngeneic tumors established in an immunocompetent host [116]. These data also remind us that using tagged antibodies to target tumor-selective antigens, together with CAR T cells against the tag, provides a regulatable therapeutic ‘switch’, since controlling the antibody dose will limit on-target–off-tumor cytotoxicity [117,118]. In an attempt to produce a CAR that can be used to treat multiple different solid tumors, Vincent et al. developed a novel probiotic-guided CAR (ProCAR) system. This system utilizes bacteria that selectively colonize tumor cores. These modified Escherichia coli release synthetic tags that are able to label tumor cells, which can then be targeted by a tag-specific CAR. The tags contain a binding domain made of the heparin-binding domain (HBD) of placenta growth factor-2, which broadly binds to collagens, fibronectins and heparan sulfate proteoglycans (HSPGs), which are abundant within solid tumors [119]. The HBD from PlGF-2 is linked to a homodimer of superfolder green fluorescent protein (sfGFP) (diGFP). Cells labeled with this tag can then be specifically targeted with a GFP-specific CAR. Human ProCARs were assessed in immunodeficient models, followed by an evaluation of murine ProCARs in immunocompetent models [119]. The use of the syngeneic models illustrated that the ProCAR system could prime the immune system to have anti-tumor responses to untagged tumor areas [119].

As mentioned, a major limitation of CAR adoptive therapy is the potential of CAR T cells to attack non-cancerous cells, known as on-target–off-tumor toxicity. To overcome this limitation, there is a need to develop technologies to terminate an active CAR response so as to limit unwanted toxicity. By the use of CAR T cells that do not directly bind to tumor cells but rather bind to ‘switches’ [117,120] that attach to the tumor cells (such as the FITC-labeled antibodies described) and by limiting or terminating the administration of the ‘switch’, it should be possible to limit the on-target–off-tumor toxicity, as mentioned in previous studies [121].

## 8. Problems with Murine CAR T Cells

As described above, the use of immunocompetent models for assessing CAR T cell efficacy is essential if we are to approach a clearer understanding of how to develop effective CAR T cell therapy of solid cancers in humans. However, it must still be acknowledged that there remain several drawbacks to the use of syngeneic mouse models for developing better CAR T cells for human therapies.

A major issue that is slowly being addressed is the poorer production of murine CAR T cells [104]. Unlike human T cells, murine T cells do not survive as well ex vivo and expand more poorly. This makes producing sufficient numbers of CAR T cells to infuse into mice very challenging. In addition, this slow rate of proliferation makes retroviral-mediated gene transfer of the CAR into the cells much harder, with lower transduction efficiencies seen in murine T cells compared to human T cells [122,123]. The inherent cytotoxic activity of murine CAR T cells also appears to be much lower than that of human CAR T cells. This can be seen by the much higher effector-to-target ratios needed to induce target cell death. In a study directly comparing the murine and human versions of an anti-GPC-1 CAR T cell, the murine version only demonstrated in vitro tumor cell lysis at 80:1 in contrast to 5:1 for the human construct. Leading on from this, it seems that even at high E:T ratios, the murine cells are less effective at lysing target cells. The absolute amount of IFNγ produced is far lower in the murine CAR T cells [61].

Another shortcoming of syngeneic models is linked to their inherent poorer survival, because they require additional cytokine support for in vivo function and persistence. It is common to administer high doses of IL-2 for several days after CAR T cell infusion, and often, multiple CAR T cell doses are required [30]. This is in contrast to the human CAR T cells, which are able to survive in immunodeficient mice with no additional support, and also differs from the clinic, where CAR T cells are normally administered without other cytokines.

Another obstacle to the translation from syngeneic CAR T mouse models to humans is the difference in the expression pattern of the target antigen between humans and mice. Additionally, some targets are not naturally expressed in mice, which makes it challenging to assess on-target–off-tumor cytotoxicity [124,125]. Although the immunocompetent syngeneic models develop tumor immune infiltrates that better mimic human tumors, studies have shown that implanted tumors can induce a greater immune response than spontaneous tumors, as the immune system does not co-evolve with the tumor, so may result in an over estimation of the anti-tumor potential of CAR T cells [126,127].

While syngeneic models can evaluate the interplay between immune cells, they cannot always adequately account for the in vivo interactions that occur in humans. Human and mouse CARs differ in their structure, as well as in their optimal activation and conditions for T cell growth and expansion in vitro and target antigen expression in normal and cancer tissues. Hence, to corroborate results, it is wise if the CAR T cells are tested in both immunodeficient and syngeneic models so as to assess the contribution of the immune cells [128].

## 9. Conclusions and Future Directions

CAR T cells have generated impressive clinical outcomes in some hematological malignancies; however, this is yet to be translated to the solid tumor field. Thus far, most pre-clinical CAR T cell models have failed to fully recapitulate the challenges faced in patients. Syngeneic models using murine CAR T cells in immunocompetent mice can overcome many of the disadvantages of current immunodeficient models and allow researchers to examine the response of the host’s innate and adaptive immune cell populations. Such studies can lead to experimental designs that target one or more host populations and, as described above, often improve CAR T cell efficacy. Thus, while it is acknowledged that mouse CAR T cells are less efficacious than human CAR T cells, it is essential that we develop better syngeneic models that possess an immunocompetent tumor microenvironment. These models must not only include immunosuppressive cells but also be able to generate positive bystander effects in the host immune system. The utilization of these models is required for the development of more effective CAR T cell combination therapies for the effective treatment of patients with solid cancers.

## Figures and Tables

**Table 1:** Summary of the murine models for CAR T cell pre-clinical testing.

	Immune System	Immune Cells	CAR T Cells	Tumor Cells	Tumor Antigens
**Xenograft**	Immunodeficient	-	Human	Human	Human
**Syngeneic**	Immunocompetent	Murine	Murine	Murine	Murine
**Transgenic**	Immunocompetent	Murine	Murine	Murine	Human
**Humanized**	Immunocompetent	Human	Human	Human	Human

## Supplementary Materials

The following supporting information can be downloaded at https://www.mdpi.com/article/10.3390/cancers16183186/s1, Table S1: Existing in-vitro studies utilizing murine CAR T cells.
